# The Association between Perceived Social Hardship and Future Orientation among Hong Kong Young People: The Mediation Role of Belief in a Just World

**DOI:** 10.3390/ijerph17144957

**Published:** 2020-07-09

**Authors:** Jian-Bin Li

**Affiliations:** Department of Early Childhood Education, The Education University of Hong Kong, Hong Kong, China; lijianbin@eduhk.hk

**Keywords:** future expectation, feelings towards the future, future planning, social hardship, belief in a just world, emerging adults

## Abstract

A positive future orientation (FO) is associated with a range of positive outcomes. It is a crucial resilience factor that assists individuals to navigate developmental difficulties during the transition to young adulthood and during periods of social adversity. Exposure to negative social context threatens young people’s FO. The social demonstrations and the outbreak of coronavirus disease in Hong Kong over the past year have caused considerable hardship to the local society. Under such circumstance, young people in Hong Kong may develop a negative FO. Scant research has directly examined the relationship between perceived social hardship and FO as well as the underlying mechanism among Hong Kong young people. In this study, we tested the idea that young people’s perceived social hardship would be negatively related to FO via belief in a just world, a well-known foundation for individuals to think, feel, and plan their future. Participants were 554 students recruited from eight universities in Hong Kong. They filled in self-report questionnaires online. Results of structural equation modeling supported our hypothesis. These findings shed light on how to nurture Hong Kong young people to develop a positive mindset during periods of social hardship.

## 1. Introduction

Future orientation (FO) refers to an individual’s attitude toward the future [1]. It becomes especially salient during late adolescence/young adulthood when the exploration of life and career becomes important [2]. Mounting evidence has suggested that young adults with a positive FO show better well-being and engage in behaviors that constructively contribute to the society, while young adults with a negative FO show poorer wellbeing and tend to engage in behaviors that may jeopardize the society’s values, economy, and public safety [3,4,5]. Not surprisingly, FO is considered a foundational aspect of youth resilience and optimal development under adversity [6,7,8].

Since the Great Recession that occurred in the late 2000 in US and many European countries, researchers increasingly recognize the importance to examine the effect of social hardship on young adults beliefs and outlooks, because exposure to social hardship (e.g., economic crisis, violence, negative geopolitical changes) may undermine an individual’s FO [9]. In addition to global changes, Hong Kong society has experienced tremendous adversity over the past year primarily due to two events. One is the social demonstrations since June 2019, and the other is the outbreak of coronavirus disease (COVID-19) since early 2020. Regarding the demonstration, the demonstrators (largely comprised of young people such as university students) have launched hundreds of protests to require the government to satisfy their demands, with deviance (e.g., vandalism) and violence (e.g., use of weapons) frequently used in many demonstrations. This event has caused a great deal of economic hardship (e.g., a rise in unemployment rate), institutional hardship (e.g., a rise in distrust in government), and social climate hardship (e.g., a rise in violent crime). For instance, the unemployment rate increased from 2.8% between April and June 2019 to 3.4% between November 2019 and January 2020 [10]; the public distrust in the Hong Kong government increased from 48.5% in the first half of 2019 to 63.9% in the second half of 2019 [11]; and the number of violent crime cases in 2019 increased by 8.4% compared to the number in 2018 [12]. The Hong Kong government has realized that in addition to structural and geopolitical reasons, young people’s negative beliefs and values about the future are one of the most deeply-rooted causes of the social demonstrations, and thus, the government has called upon an urgent investigation to nurture young Hong Kong people to develop a positive sense about the future while studying policies that would address the structural and geopolitical causes of the demonstration, with the aim to facilitate social reconciliation [13]. Regarding the outbreak of COVID-19, the Hong Kong government has actively adopted a number of measures (e.g., shutting down the borders, implementing compulsory 14-day quarantine when traveling to Hong Kong, and suspending schools for a significant period). While these measures are largely effective, they have also exacerbated the existing economic, institutional, and social climate hardship caused by the demonstrations. For instance, the unemployment rate continued increasing to 5.9% between March and May 2020 [10]; the distrust in government also raised slightly to 64.8%, reaching the highest since Hong Kong’s return in 1997 [11], and the number of violent crime cases in the first quarter of 2020 increased by 7.4% compared to the number in the first quarter of 2019 [12].

So far, little research has examined Hong Kong young people’s FO under the influence of social hardship and its underlying mechanisms. In this study, we assume that an individual’s perception of social hardship would be negatively related to FO via belief in a just world, a well-known foundation for people to think, feel, and plan for their future [14,15]. We tested this idea among Hong Kong young people. 

### 1.1. Social Hardship and Future Orientation

FO is a multidimensional construct that comprises cognitive, affective, and motivational components, including the ability to imagine one’s future life circumstance (e.g., future expectations), the extent to which one is optimistic or pessimistic about the future (e.g., feelings towards the future), and the extent to which one engages in goal setting or planning (e.g., future planning) [2]. According to the socio-ecological framework of FO [16], negative social context restricts young people’s FO by constraining their fulfillment of developmental tasks. By comparing cohort data collected before and after the occurrence of social hardship, existing research revealed that exposure to social hardship (e.g., economic downturn and social violent climate) reduces young people’s future expectation [17,18]. Despite their importance, these studies treated social hardship as a “natural experiment” rather than considered it as individual differences. However, even though all young people are exposed to the social hardship, they may develop different perception of how severe the social hardship is, and thus, they are supposed to be affected differentially by the hardship. In this sense, directly measuring one’s perceived social hardship (PSH) would allow a more nuanced examination of the relationship between social hardship and FO. To fill this gap, we measure PSH in this study.

We define PSH as an individual’s evaluation about the severity of hardship a society has undergone. This perception refers to people’s sense of hardship at the societal level, and it should be closely (although not perfectly) tied to actual social hardship. Logically, people’s PSH should reflect the actual situation. In other words, the more severe actual social hardship is, the stronger people’s PSH would be. In this sense, the major source of variation of PSH would come from actual social hardship. Three types of hardship are particularly harmful because they are closely connected to citizen’s security and lives, including institutional, economic, and social climate hardship. For instance, institutional hardship comprising non-transparent procedure, regulatory hindrance, inadequate and uncertain policies, weak legal protection, restricted freedom and rights, and poor public services may hamper citizens in obtaining sufficient sociopolitical and economic resources to survive and grow [19]. If a society experiences economic hardship (e.g., economic downturn, shrinking income), it may dispose citizens to be economically disadvantaged in procuring basic economic materials to survive and thrive. If a society experiences social climate hardship (e.g., social unsafety, social instability), citizens may feel their surroundings insecure and feel fear at the social level. The consequences of such social hardship may result in a fragmentation of human lives and in the increase of difficulties for young people to think about and plan their future [9,20]. Hence, we expect that young people who perceive severe PSH would be less oriented to the future.

### 1.2. The Mediation of Belief in a Just World

Belief in a just world (BJW) refers to an individual’s faith that they can obtain what they deserve in a fair world [21]. BJW is divided into personal and general BJW based on whom is the recipient of the justice. The personal BJW reflects the beliefs that, overall, events in one’s life are fair, while the general BJW reflects the belief that, basically, the world is a just place [14]. Prior research found that personal BJW is related to mental health more strongly than general BJW in WEIRD (western, educated, industrialized, rich and democratic) samples but that general BJW is more related to mental health among people in developing countries (e.g., China) and promote resilience among people confronting adversities [22]. Hence, in this study, we focused on general BJW (hereafter BJW). We expect that BJW would mediate the relationship between PSH and FO.

First, according to Lerner and Miller [23], individuals are motivated to believe in a just world where people generally get what they deserve and deserve what they get; this belief enables people to confront their physical and social environment as though it were stable and orderly and serves important adaptive functions. Without such a belief, it would be difficult for the individual to commit themselves to the pursuit of long-term goals. BJW endows individuals with the confidence that they will be treated fairly by others and will not fall victim to an unforeseeable disaster, which is the foundation for people to invest their future because they are confident that their investment will be fairly rewarded [14]. In this sense, individuals with high levels of BJW tend to be more orientated to the future. Prior research has found a positive relationship between BJW and FO among young people [15,22].

Second, being confronted with injustice, either observed or experienced, threatens the belief that justice prevails in the world [14]. Moreover, repeated, long-term, or large-impact negative events can weaken an individual’s BJW [24]. For instance, individuals who are unemployed for a long time report less BJW than those who are only momentarily unemployed [25]. In addition, young people exposed to school violence show poor BJW [26]. A recent study also found that young people who experienced significant negative life events had lower levels of BJW [21]. The association between negative social context and BJW could be particularly salient among young people, because late adolescence/young adulthood (18–25 years old, [27]) is arguably the most formative period regarding the development of beliefs about how the society, politics, and the economy are shaped, and it is commonly assumed that the beliefs and values that crystallize during this period remain largely stable throughout the lifespan [28]. In this sense, young people who perceive high levels of social hardship are likely to have low levels of BJW. Thus, we expect that PSH would be negatively related to BJW among young people.

Collectively, young people who perceive high level of PSH would tend to believe the world is less just; without such a confidence that their investment in the future would be fairly rewarded, they will show less future orientation. Therefore, we expect that BJW would mediate the association between PSH and FO.

### 1.3. The Current Study

**Figure 1:**
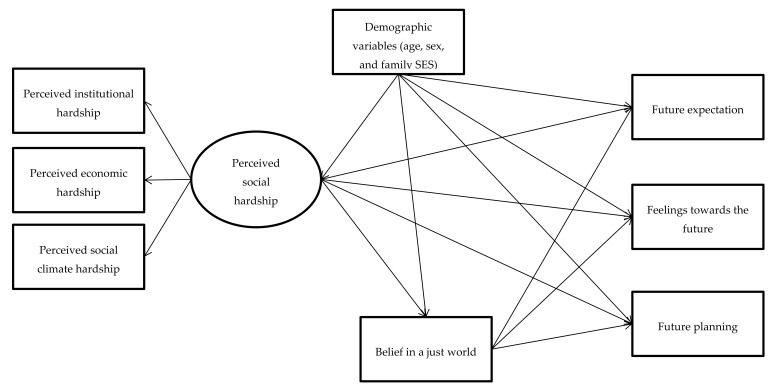
Conceptual mediation model of belief in a just world in the association between perceived social hardship and future orientation.

In this study, we examined the association between PSH and various FO components (i.e., future expectations, feelings towards the future, and future planning) and the mediation effect of BJW among young people in Hong Kong. In addition, since prior early research has found that sex, age, and family socioeconomic status (SES) are related to FO [29,30], we also control for these factors when addressing the research questions (Figure 1).

## 2. Methods

### 2.1. Participants and Procedure

Participants were 554 students from eight government-funded universities in Hong Kong (62.1% females, 81.7% studying for a bachelor’s degree, age = 21–40 years, SD = 3.47). The data were collected in May 2020. To recruit participants, research assistant and student helpers posted a survey link on the forums and social media groups of each targeted university. We also invited colleagues working in these universities to distribute the survey link via their internal e-mails. A total of 782 students visited the website, and 554 provided complete data. It should be noted that these online recruited samples may be non-random in nature because students who frequently checked their e-mails and surfed the social media groups/forums had higher likelihood to know and join this survey than those who did not. This study was reviewed and approved by the Education University of Hong Kong (2019-2020-0228). All participants provided electronic consent prior to participation and received HK$100 supermarket coupon upon completion.

### 2.2. Measures

#### 2.2.1. Perceived Social Hardship

We created a Perceived Social Hardship Scale to measure participants’ perception of how much hardship Hong Kong society had undergone in different aspects over the past year. We listed 13 aspects frequently mentioned by government and social media, covering three domains including institutional (5 items: political freedom, legal justice, human rights, freedom of the press, and freedom of voice), economic (4 items: economic environment, employment rate, citizens’ income and citizens’ housing burden) and social climate (4 items: social safety, social stability, social equality, and social justice) hardship. We conducted a confirmatory factor analysis to examine if these respective items would load on the planned domains as well as to investigate if the three first-order latent constructs would load on a second-order construct (i.e., PSH). The model fit of a second-order model was good with three pairs of residuals correlated due to homogeneity in item meanings (i.e., freedom of press and freedom of voice, economic environment and employment rate, social safety and social stability (χ2(59) = 177.04, RMSEA = 0.06, CFI = 0.96, TLI = 0.94). All item loadings were above 0.60, and all the first-order constructs had high loadings on the second-order construct (>0.60). All items are rated on a five-point scale (from “1 = not at all” to “5 = very much”). A higher mean score indicates participants perceived that Hong Kong society had undergone more hardship. The internal reliability was 0.93 for perceived institutional hardship, 0.86 for perceived economic hardship, and 0.85 for perceived social climate hardship.

#### 2.2.2. Belief in a Just World

We used the General Belief in a Just World scale [31] to measure the extent to which participants believe the world he is living in is just. The scale consists of 6 items rated on a six-point scale (from “1 = strongly disagree” to “6 = strongly agree”). We translated this scale into Chinese following a back-translation procedure [32]. A sample item is “I think the world is basically a just place.” A higher mean score indicates participants tended to believe the world is just. The internal reliability of the total scale was 0.85.

#### 2.2.3. Future Orientation

We used different scales to measure the three FO components, and we translated these scales into Chinese following a back translation procedure [32]. To measure future expectation, we used the Future Expectation Scale [33]. This scale consists 8 items rated on a five-point scale (from “1 = very low” to “5 = very high”), with a higher mean score indicating stronger expectation towards the future. A sample item is “What is the chance that you will have a job that pays well?” The internal reliability of this scale was 0.86. To measure feelings towards the future, we used the Future Emotion Questionnaire [34]. This scale consists of 7 items rated on a six-point scale (from “1 = not at all” to “6 = very much”), with a higher mean score indicating more positive feelings towards the future. A sample item is “I feel confident about the future.” The internal reliability of this scale was 0.89. To measure future planning, we used the Future Planning Scale used in the YouTH got talent project [35]. This scale consists of 3 items rated on a six-point scale (from “1 = strongly disagree” to “6 = strongly agree”), with a higher mean score indicating that participants started to plan and have implemented the plan for the future. A sample item is “I have plans for what I am going to do in the future.” The internal reliability of this scale was 0.83. 

#### 2.2.4. Demographic Variables

We also collected participants’ demographic variables, including sex (0 = male, 1 = female), age, and both parents’ monthly income. We standardized and averaged parents’ monthly income to represent participants’ family SES.

### 2.3. Data Analysis

We analyzed the data with SPSS 18.0 (SPSS Inc., Chicago, IL, USA) and Mplus 7.0 (Muthén & Muthén, Los Angeles, CA, USA) with 0.05 as the significance level throughout. First, we conducted descriptive statistics to examine the individual differences in the levels of PSH, BJW, and FO. Second, we performed Pearson correlation analyses to examine the bivariate associations between PSH, BJW, and FO. Third, we carried out structural equation modeling (SEM) to examine the association between PSH and FO as well as the mediation effects of BJW, controlling for participants’ sex, age, and family SES. Values of RMSEA, CFI, and TLI were used to evaluate the model fit. The model fit would be deemed to be acceptable if the value of RMSEA was not greater than 0.08 and the values of CFI and TLI were not less than 0.90 [36,37]. Since bootstrapping technique has higher statistical power than traditional methods such as Sobel’s test [38], we employed bootstrapping technique (N = 10,000) and its 95% confidence interval (CI) to determine the significance of the mediation effect. A significant mediation effect would be deemed to be present if the 95% CI does not include zero.

## 3. Results

### 3.1. Descriptive Statistics and Bivariate Correlations

As shown in Table 1, participants reported high levels of institutional (4.21 out of 5), economic (4.00 out of 5) and social climate (4.33 out of 5) hardship, medium level of future expectation (3.26 out of 5) and future planning (3.97 out of 6) as well as relatively low levels of positive feelings towards the future (3.10 out of 6) and BJW (3.26 out of 6). Results of correlation analysis showed that PSH components were negatively and significantly associated with BJW, future expectation, and feelings towards the future, while BJW was positively and significantly related to all FO components.

### 3.2. The Relationship Between PSH and FO and the Mediation of Belief in a Just World

**Figure 2:**
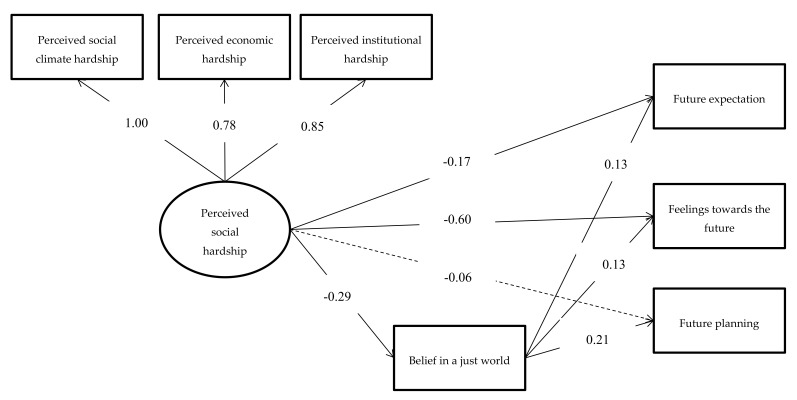
Mediation model of belief in a just world in the association between perceived social hardship and future orientation. Note: the associations between demographic variables and perceived social hardship, belief in a just world, and the three future orientation components were controlled for and presented in Table 2, but they are not shown in the figure for simplicity. Solid line indicates significant paths; dash line indicates insignificant paths.

The model fit was acceptable, χ2(14) = 42.40, *p* < 0.001, RMSEA = 0.06, CFI = 0.98, TLI = 0.93. In addition, as shown in Table 2, the model explained 12.6%, 23.7%, and 8.3% variance of future expectation, feelings towards the future, and future planning, respectively. The factor loadings and the path coefficients are summarized in Figure 2. Moreover, after controlling for demographic variables, high levels of PSH were negatively and significantly associated with future expectation (B = −0.17, SE = 0.06, *p* = 0.006) and feelings towards the future (B = −0.60, SE = 0.07, *p* < 0.001), but not with future planning (B = −0.06, SE = 0.08, *p* = 0.501). More importantly, as shown in Table 3, results of mediation analyses found that BJW significantly mediated the “PSH and future expectation” (B = −0.04, SE = 0.01, *p* = 0.005, 95% CI = [−0.07, −0.02]), the “PSH and feelings towards the future” (B = −0.04, SE = 0.02, *p* = 0.023, 95% CI = [−0.08, −0.01]), and the “PSH and future planning” (B = −0.06, SE = 0.02, *p* = 0.005, 95% CI = [−0.11, −0.03]) associations, as the 95% CI of each mediation estimate did not include zero. Regarding the effect sizes of these mediation effects, Kenny proposed that the standardized mediation coefficient of 0.01, 0.09, and 0.25 represents small, medium, and large effect sizes, respectively [39]. Based on these criteria, our results suggested that all the three mediation effects at least reached small effect size, thus indicating that these mediation effects were nontrivial.

In addition, we examined whether our sample size was sufficient to detect the mediation effect by calculating the sample size needed to achieve a priori power of 0.80 based on the current findings. We conducted the analysis at: https://davidakenny.shinyapps.io/MedPower/. We found that 272, 356, and 254 participants were needed to detect a mediation effect for future expectation, feelings towards the future, and future planning, respectively. Given that our sample size was 554, we deemed that our study had enough number of participants to achieve a priori power of 80 in detecting a mediation effect.

## 4. Discussion

The on-and-off social demonstrations since June 2019 and the outbreak of COVID-19 since early 2020 have caused considerable adversity to Hong Kong society. Young people, especially university students, are active in social demonstrations partly because this is one of the many forms of students’ civic engagement and citizenship as taught by higher education [40]. Nevertheless, university students are also particularly vulnerable to the social hardship (especially economic hardship) induced by social demonstrations as well as COVID-19 because such hardship imposes great challenges for students to fulfill their developmental tasks as emerging adults such as entering the labor market and becoming financially independent. In this study, we examined young people’s perception of social hardships primarily induced by these events, associated it with their future orientation, and investigated BJW as a potential underlying mechanism. Several important results are generated and commented on below.

We found that young people reported high levels of PSH, especially social climate hardship. These perceptions are in accordance with actual social hardship, such as the rise in distrust of institutions [11], unemployment rate [10], and violent crime over the past year in Hong Kong [12]. The scores also showed considerable individual differences, with some perceiving no hardship at all while others perceiving extreme hardship. One possible interpretation is that since PSH should be related to actual social hardship; some people may possess more resources to navigate the influence of actual social hardship on them, and therefore, they tend to perceive lower PSH than others who have fewer resources. This explanation is consistent with Lazarus’ cognitive appraisal theory in that one’s risk perception depends on both the actual environmental risks and the resources that can be negotiated to deal with the risks [41]. For instance, the current findings reveal that participant’s family SES was negatively related to PSH, which suggests that one’s own economic resources could be an important factor that intervenes with actual social hardship to shape one’s PSH. These findings reaffirm the necessity of measuring individual differences in perceived social hardship, rather than treating it as a “natural experiment” and considering the negative social context imposing similar impacts on everyone as prior research did [18].

In addition, we found that participants reported relatively low levels of belief in a just world, future expectation, and positive feelings towards the future and medium levels of future planning. These descriptive data inform that under the influence of social demonstrations and the outbreak of COVID-19, Hong Kong young people’s BJW and FO are poorly developed. Despite the fact that these findings may reflect a snapshot of the current situation, research about resilience suggests that individuals’ perception of risk and hardship and their adjustment outcomes may change as the events that cause the risk and hardship unfold [42]. In this sense, future research should continuously monitor these issues, since the social demonstrations still occur from time to time, and the policies to control the COVID-19 pandemic are still in effect at the expense of economy.

Prior research primarily treated social hardship as a “natural experiment” and revealed that social hardship undermines young people’s FO [18]. This study directly measured the individual differences in PSH and associated it with FO among young people. Consistent with prior findings, this research generally found that individual differences in PSH were negatively related to FO. Specifically, PSH was directly related to less future expectation and positive feelings towards the future but not to future planning. Nevertheless, results of mediation analyses found that PSH was associated with all the three FO components through BJW. That is, young people who perceived high levels of PSH tend to believe the world is less just which further associated with less orientation towards the future. These findings are in accordance with prior research which discloses that an individual’s belief serves as a mechanism between ecological context (e.g., family) and FO [43,44]. Such findings also support the socio-ecological framework of FO, which proposes that an individual’s belief would play a key role in the influence of social-ecological systems on the development of FO [16]. This study goes beyond past research in that this research not only considers the individual differences in PSH (rather than treating it as a natural experiment), but it also reveals an underlying mechanism that explains how PSH is associated with FO.

This study bears both theoretical and practical implications. Theoretically, FO becomes particularly salient among late teens/young adulthood [2], and the social-ecological framework of FO suggests that FO evolves by continuously interacting with the social environment [16]. However, past research mainly examined FO among young adolescents and focuses on the role of microsystem contexts such as family and school [45,46,47]. Although a few studies have examined the role of macro social context in young adults’ FO, they failed to consider the individual differences in the influence of social context [18]. This study contributes to a greater understanding of the association of negative social context on young people’s FO and its underlying mechanisms by measuring PSH and conducting the study among university students. Practically, the Hong Kong government has called upon investigation to nurture young people to develop a positive mindset to facilitate social reconciliation [13]. The first step to do so is to understand the precursors of young people’s FO and how the precursors operate. In light of the present findings, enhancing young people’s BJW might be an important avenue to boost their FO, especially for those who perceive high levels of PSH. Although there are few intervention programs that directly improve young people’s belief in a just world, some indirect measures can be employed for this purpose. Prior research found that social support and social trust are two fundamental pillars of beliefs in a just world [48], which implies that practitioners might possibly strengthen young people’s belief in a just world by enhancing these two pillars. For instance, teaching young people the history of the local society appears to be useful in improving social trust [49]. In addition, we also need to point out that although PSH refers to an individual’s perception of social hardship, we do consider that one’s perceived social hardship should largely align with the actual social hardship. In this sense, besides enhancing young people’s FO, it is also important for the government to directly address the actual social hardship (e.g., reducing the unemployment rate and violent crime as well as increasing people’s trust in government) in order to better cultivate young people’ positive mindset and to facilitate social reconciliation.

This study is not without limitations. First, only self-report questionnaires were used, and this may cause common method bias and the cross-sectional design may limit causal inference. We recommend that readers should see the present findings as preliminary and interpret the findings with caution. Nevertheless, given the urgent situation in Hong Kong society, procuring local data and informing the government and scholars about young people’s PSH and FO is important, necessary, and timely. Nevertheless, we need to emphasize again that these findings may be restricted to the current periods of social adversity in Hong Kong and are not readily generalizable to other periods. We encourage future study to employ more sophisticated design (e.g., longitudinal design) and multiple informants to continuously examine the dynamic association between PSH and FO as the social situation unfolds, with the hope to benefit a positive development among young people in Hong Kong during the periods of social adversity. Second, this study only focused on university students and the samples contained more females than males. In addition, the sample of this study was non-random, with some students being more likely to participate in the current study than others. All of these limit the representativeness of the young people of Hong Kong. Future research could consider recruiting samples with more diverse demographic backgrounds to enhance the generalizability of the findings.

## 5. Conclusions

In conclusion, this study examined the association between perceived social hardship and future orientation as well as investigated the mediation effect of belief in a just world among Hong Kong young people. We found that young people reported high levels of perceived social hardship and relatively low levels of future expectation and positive feelings towards the future. Moreover, results of structural equation modeling suggested that high levels of perceived social hardship were related to lower future orientation both directly and indirectly through young people’s belief in a just world. We believe that these findings have important implications for enhancing Hong Kong young people’s positive mindset during the periods of social adversity.

## Figures and Tables

**Table 1:** Descriptive statistics and bivariate correlations.

	Min	Max	M	SD	1	2	3	4	5	6	7
1. Perceived institutional hardship	1.00	5.00	4.21	0.89	-						
2. Perceived economic hardship	1.00	5.00	4.00	0.80	0.48 **	-					
3. Perceived social climate hardship	1.00	5.00	4.33	0.73	0.61 **	0.56 **	-				
4. Belief in a just world	1.00	6.00	3.26	0.98	−0.16 **	−0.10 *	−0.09 *	-			
5. Future expectation	1.00	5.00	3.12	0.67	−0.21 **	−0.10 *	−0.09 *	0.26 **	-		
6. Feelings towards the future	1.00	6.00	3.10	0.94	−0.43 **	−0.26 **	−0.29 **	0.29 **	0.61 **	-	
7. Future planning	1.00	6.00	3.97	0.97	−0.12 **	−0.03	−0.01	0.25 **	0.62 **	0.47 **	-

Note: * *p* < 0.05; ** *p* < 0.01. Min = minimum score; Max = maximum score; M = mean; SD = standard deviation.

**Table 2:** The associations among perceived social hardship, belief in a just world, and future orientation.

	B	SE	*p*	95% CI
PSH as outcome (*R*^2^ = 1.4%)				
Age	0.00	0.01	0.898	[−0.02, 0.02]
Sex	0.03	0.04	0.432	[−0.07, 0.12]
Family SES	−0.08	0.04	0.039	[−0.15, −0.01]
				
BJW as outcome (*R*^2^ = 8.4%)				
Age	0.02	0.02	0.195	[−0.01, 0.05]
Sex	−0.02	0.11	0.846	[−0.09, 0.27]
Family SES	−0.23	0.07	0.001	[−0.36, −0.09]
PSH	−0.29	0.08	<0.001	[−0.43, −0.14]
				
Future expectation as outcome (*R*^2^ = 12.6%)				
Age	0.04	0.01	<0.001	[0.02, 0.06]
Sex	−0.02	0.04	0.597	[−0.14, 0.03]
Family SES	0.04	0.04	0.318	[−0.03, 0.10]
PSH	−0.17	0.06	0.006	[−0.29, −0.05]
BJW	0.13	0.03	<0.001	[0.07, 0.20]
				
Feelings towards the future as outcome (*R*^2^ = 23.3%)				
Age	0.03	0.02	0.049	[0.00, 0.06]
Sex	0.11	0.07	0.109	[−0.07, 0.17]
Family SES	0.03	0.06	0.548	[−0.07, 0.14]
PSH	−0.60	0.07	<0.001	[−0.74, −0.47]
BJW	0.13	0.05	0.004	[0.04, 0.22]
				
Future planning as outcome (*R*^2^ = 8.3%)				
Age	0.04	0.01	0.003	[0.01, 0.07]
Sex	0.03	0.12	0.795	[−0.27, 0.09]
Family SES	0.10	0.05	0.066	[−0.00, 0.21]
PSH	−0.06	0.08	0.501	[−0.22, 0.11]
BJW	0.21	0.05	<0.001	[0.12, 0.30]

Note: PSH = perceived social hardship; BJW = belief in a just world; SES = socioeconomic status; CI = confidence interval.

**Table 3:** Summary of the mediation effects of belief in a just world in the association between perceived social hardship and future orientation.

Indirect Paths	B	SE	*p*	95% CI	β
PSH → BJW → Future expectation	−0.04	0.01	0.005	[−0.07, −0.02]	−0.04
PSH → BJW → Feelings towards the future	−0.04	0.02	0.023	[−0.08, −0.01]	−0.03
PSH → BJW → Future planning	−0.06	0.02	0.005	[−0.11, −0.03]	−0.04

Note: PSH = perceived social hardship; BJW = belief in a just world. B refers to unstandardized mediation effect; SE refers to standard error of the unstandardized mediation effect; CI refers to confidence interval; β refers to standardized mediation effect.
